# *Strongyloides* Infection Among Patients at a Long-Term Care Facility — Florida, 2010–2012

**Published:** 2013-10-25

**Authors:** Andrea Leapley, Alazandria Cruze, Alvaro Mejia-Echeverry, Danielle Stanek, Elesi Quaye, Stephany Vento, Isabel McAuliffe, Susan P. Montgomery, Aaron M. Samuels

**Affiliations:** Florida International Univ, Miami; Florida Dept of Health Miami-Dade County; Bureau of Disease Control and Prevention; Bureau of Public Health Laboratories-Miami, Florida Dept of Health; Div of State and Local Readiness, Office of Public Health Preparedness and Response; Div of Parasitic Diseases and Malaria, Center for Global Health, CDC

During a 2-week period in August 2011, two patients in a long-term care facility in Miami-Dade County, Florida, had gastrointestinal symptoms; microscopic examination of stool specimens showed that both harbored *Strongyloides stercoralis*, an intestinal nematode. A subsequent chart review revealed an additional case within the facility 1 year earlier. Concerned about the possibility of an outbreak, the associate director of patient care services at the facility contacted the Florida Department of Health in Miami-Dade County and the Florida State Department of Health, which contacted CDC. This report describes the subsequent investigation.

In May 2012, a serologic and risk-factor survey of residents and staff was performed to assess the prevalence of and associations with infection. *Strongyloides* informational packets were distributed to all residents and staff members, and consent for serologic testing was obtained. In June, blood samples from consenting residents and staff members were tested for *S. stercoralis*–specific antibody testing by crude antigen enzyme-linked immunosorbent assay. This serologic test becomes positive after infection (how long after infection is not well defined), and antibody titers typically drop to <50% by 6–18 months after successful treatment of the parasite.

In a convenience sample of 106 of the 176 facility residents, 12 (11%) had a positive result, as did three from a convenience sample of 26 of the 238 staff members. All 15 persons with positive results reported being born either in North America (five) or the West Indies (10). Thirty-seven long-term care facility residents in the convenience sample were born in the United States or Mexico, and four (10.8%) had results positive for *S. stercoralis*-specific antibody; only one of these persons reported no travel outside of the United States. Six long-term care facility residents reported corticosteroid use in the last 3 months, and none were infected. Because no prior testing had been performed, assessing whether any of the infections had been acquired within the facility was not possible.

Recommendations were made to offer testing and treatment to the residents and staff members who had not yet been approached and to extend this offer to incoming residents. Further research is needed to determine the prevalence of *Strongyloides* infection and the risk for transmission to help inform screening strategies for long-term care facilities.

